# In-sensor neural network for high energy efficiency analog-to-information conversion

**DOI:** 10.1038/s41598-022-23100-4

**Published:** 2022-10-29

**Authors:** Sudarsan Sadasivuni, Sumukh Prashant Bhanushali, Imon Banerjee, Arindam Sanyal

**Affiliations:** 1 Electrical Engineering, University at Buffalo, Buffalo, 14260 USA; 2 School of Electrical, Computer and Energy Engineering, Arizona State University, Tempe, 85287 USA; 3 Mayo Clinic, Phoenix, 85054 USA

**Keywords:** Electrical and electronic engineering, Electrodiagnosis

## Abstract

This work presents an on-chip analog-to-information conversion technique that utilizes analog hyper-dimensional computing based on reservoir-computing paradigm to process electrocardiograph (ECG) signals locally in-sensor and reduce radio frequency transmission by more than three orders-of-magnitude. Instead of transmitting the naturally sparse ECG signal or extracted features, the on-chip analog-to-information converter analyzes the ECG signal through a nonlinear reservoir kernel followed by an artificial neural network, and transmits the prediction results. The proposed technique is demonstrated for detection of sepsis onset and achieves state-of-the-art accuracy and energy efficiency while reducing sensor power by $159\times$ with test-chips prototyped in 65 nm CMOS.

## Introduction

Radio frequency (RF) transmission is the largest contributor of wireless sensor power, and hence, local in-sensor signal processing is preferred to continuous RF transmission^1^, especially for bio-medical sensors since bio-medical signals, such as ECG, are naturally sparse and are usually sampled at a rate far exceeding their information rate. As an example, a low-power MedRadio transmitter consumes $67\mu$W power^2^ which is significantly higher than on-chip feature extraction^1,3^. While there are several techniques to compress RF transmission^1,4–9^, the compression ratio is typically limited to $<20\times$. Approaches to compress RF transmission so far have been sparsity-based data compression algorithms^4–6^, derivative-based adaptive sampling^7^, level-crossing sampling^8^, and adaptive resolution digitization^1,9^. The aforementioned techniques have reported compression of transmission data by $2\sim 16 \times$. In contrast to the prior techniques, we propose to embed AI in the sensor itself to analyze each ECG segment and transmit only prediction score instead of ECG data or extracted features to reduce RF transmission by $>5000\times$.

It is challenging to design in-sensor neural networks with low energy consumption since artificial intelligence (AI) algorithms are computationally intensive. The majority of attempts^10–17^ to reduce energy consumption of AI circuits use (a) in-memory/near-memory computing (b) reduced precision computations. To address this energy bottleneck in wireless bio-medical sensors, we propose an analog signal processing neural network that directly processes analog ECG samples. The key contribution of this work is design and demonstration of an on-chip analog classifier comprising of a reservoir-computer (RC) followed by a 3-layer artificial neural network (ANN) that process analog ECG segments while reducing energy consumption by $13\times$ compared to digital baseline (front-end ADC followed by digital ANN) and reduces overall sensor energy by $159\times$ compared to direct transmission of digitized ECG segment. In-sensor processing AI circuits have been demonstrated primarily for CMOS image sensing applications by performing on-chip feature extraction^18–20^ to reduce the amount of data that needs to be transmitted off-sensor. To the best of our knowledge, our work presents the first in-sensor AI circuits for analog-to-information conversion for significantly reducing transmission energy in wearable physiological sensors and extend the battery life of such sensors.

**Figure 1:**
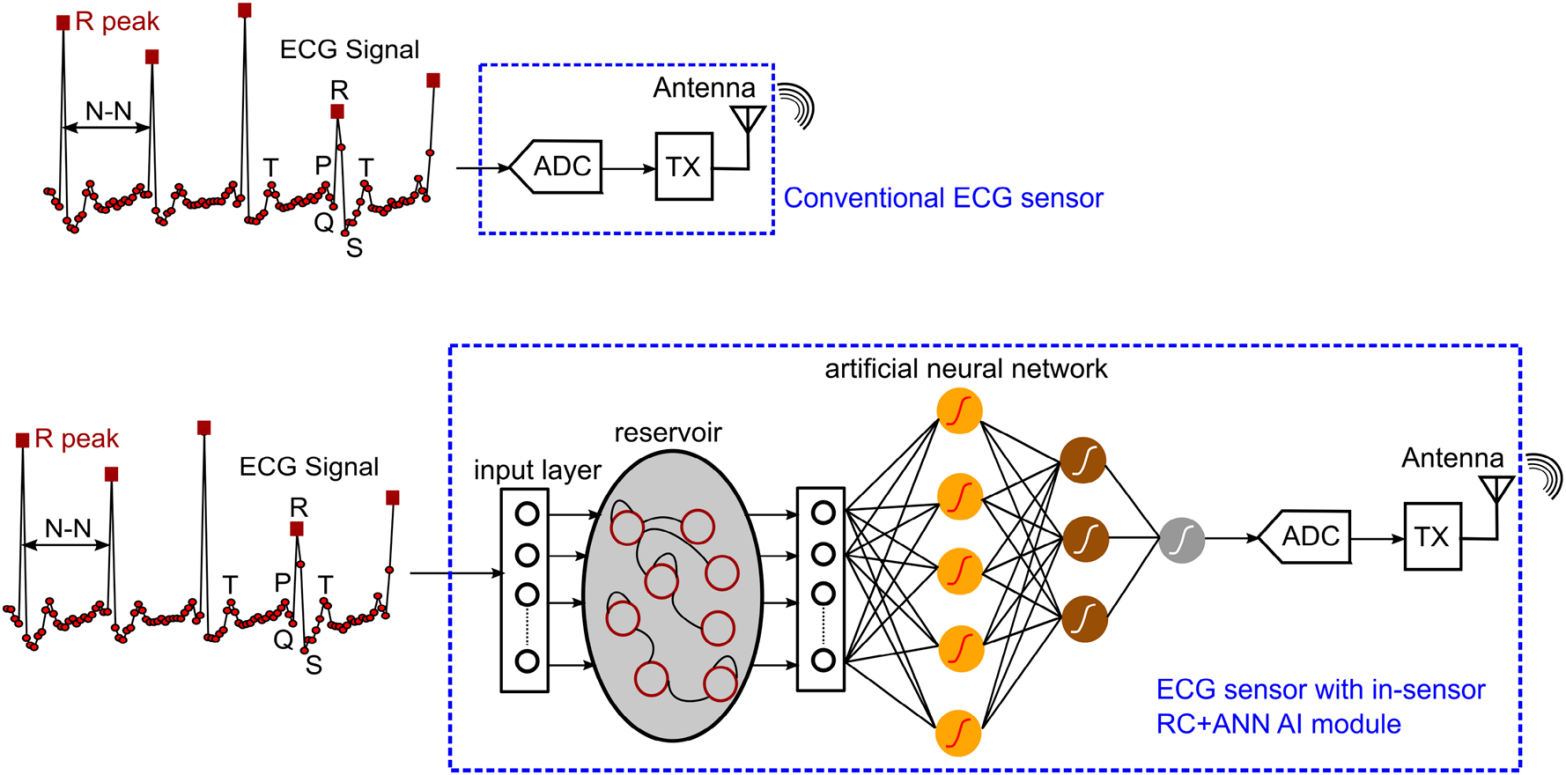
Overview of conventional ECG sensor compared to the proposed ECG sensor with in-sensor RC+ANN AI module for analyzing ECG segments and transmitting prediction score instead of all ECG samples.

Figure 1 summarizes the analog-to-information methodology of this work. In a conventional ECG sensor, all ECG samples are digitized by analog-to-digital converter (ADC) and transmitted wirelessly. In contrast, we propose in-sensor AI (RC+ANN) that analyzes non-overlapping ECG segments and prediction score of the model is digitized and transmitted wirelessly. Thus, the RF transmission volume can be reduced by a large factor which is proportional to the number of samples in the ECG segments. To ensure that the in-sensor AI does not consume large energy and offset the advantages of reduced RF transmission, we propose hyper-dimensional computing using reservoir-computing paradigm that nonlinearly projects the input sensor data to a different hyper-plane for easy separation of distinct classes in the input signal by a read-out layer. The input and reservoir layers are not trained, and nonlinearity in analog computing is leveraged to create the nonlinear kernel in the reservoir layer. In conventional analog computing nonlinearity is typically a limiting factor necessitating the analog circuits to run from high supply voltage and consume large power, thus trading off power efficiency for linearity. In contrast, analog circuits in the reservoir computer can be nonlinear and tolerate errors from incomplete settling, low gain and bandwidth. Thus, the in-sensor AI circuits can achieve high power efficiency as demonstrated in the Results section.

## Results

**Figure 2:**
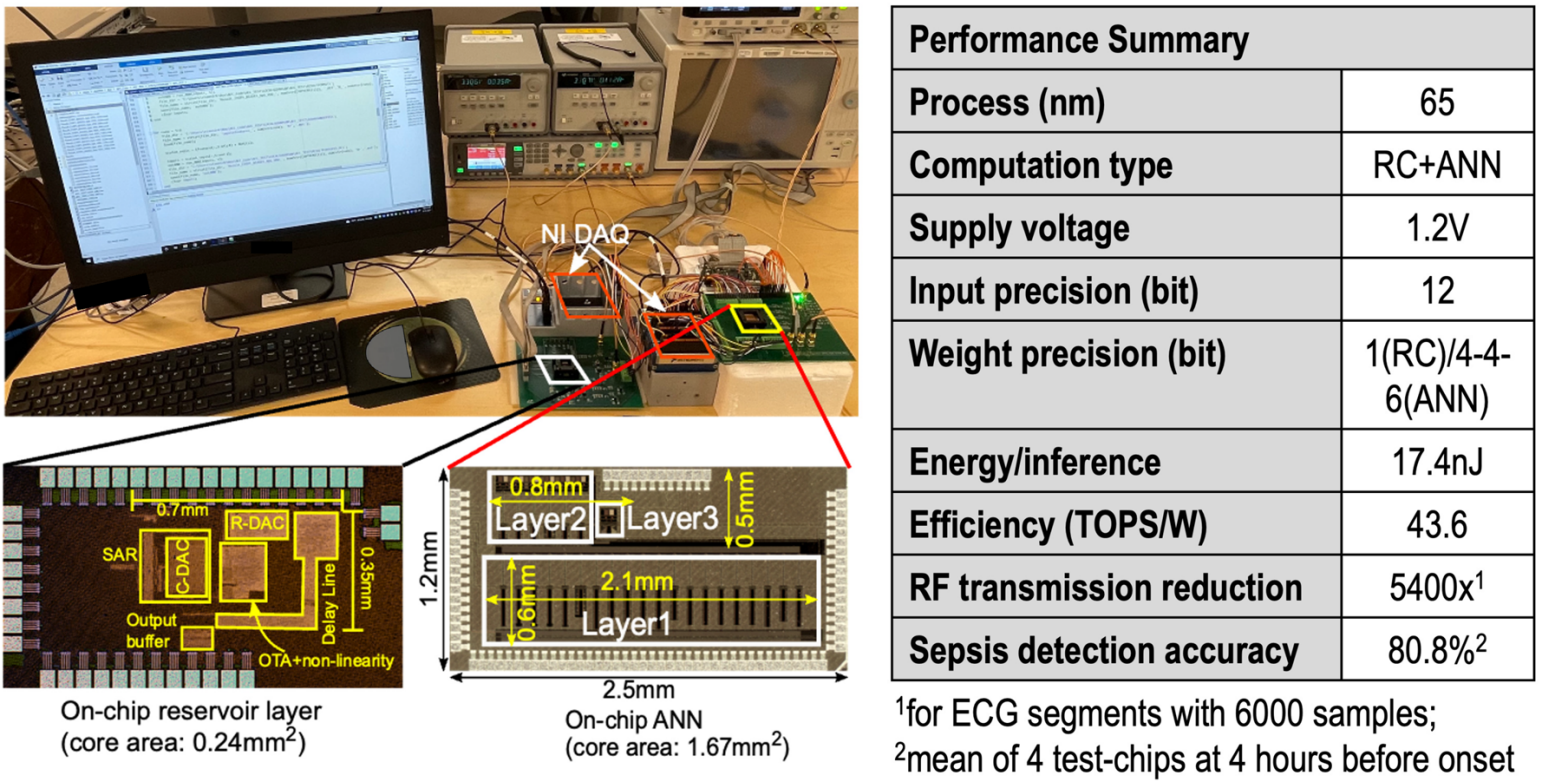
Lab measurement setup with die microphotograph of reservoir and ANN test-chips and performance summary.

The ANN and reservoir test-chips are fabricated separately in 65 nm CMOS, and integrated on printed circuit board level for lab measurement. National Instrument data acquisition (DAQ) module is used to load input data from a computer, and output of the ANN is captured using an oscilloscope and sent to the computer. A Matlab interface is used for communication between the computer, test chips and the NI DAQ. Figure 2 shows the measurement setup for the test-chips and a summary of the chip performance. The ANN has a core area of 1.67 mm$^2$ and the RC has a core area of 0.24 mm$^2$. The on-chip reservoir layer consumes 2 nJ/inference and the ANN consumes 7 nJ/inference while the off-chip reservoir input matrix multiplier consumes 8.4 nJ/inference at 1.2 V supply and operating at $F_s=1$ kHz. The energy for communication between the test chips is not included since this will be amortized once the two chips are integrated on the same die.

The proposed technique is demonstrated on the publicly available MIMIC–III dataset which comprises of de-identified, comprehensive clinical data of patients admitted to the Beth Israel Deaconess Medical Center in Boston, Massachusetts. The MIMIC–III dataset has 4559 patients with 40.2% sepsis patients and 59.8% non-sepsis patients, male/female split for sepsis and non-sepsis patients of 53.8%/46.2% and 55.8%/44.2% respectively. The dataset is randomly partitioned into 80% training samples and 20% test samples. ECG segments with 6000 samples are used for detection of sepsis 4 h before onset. All methods were carried out in accordance with relevant guidelines and regulations (Declaration of Helsinki).

**Figure 3:**
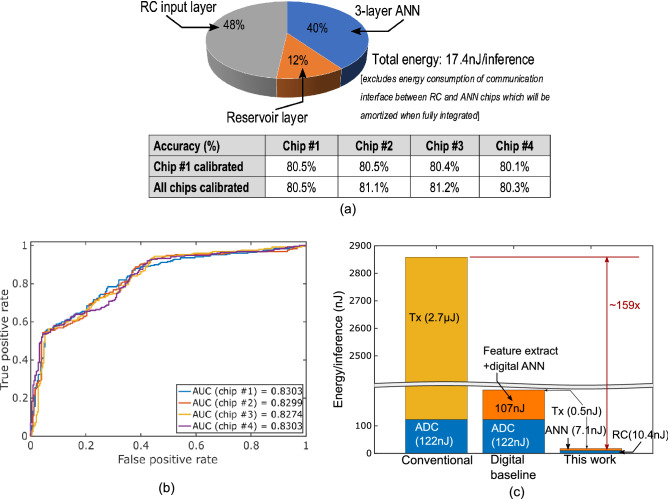
(**a**) Energy breakdown and measured accuracy of 4 test chips; (**b**) AUROC plots for 4 test-chips and; (**c**) comparison of energy/inference with conventional sensor technique of transmitting all digitized data and digital baseline.

Figure 3a shows energy breakdown and measured performance metrics for 4 test-chips. Due to offset induced by random mismatches introduced during chip fabrication, the decision threshold for each chip is shifted after fabrication. To correct for the offset, each chip is calibrated by applying the training samples to the test-chip and setting the decision threshold voltage to maximize prediction accuracy on training samples. The proposed RC+ANN combine detects sepsis with mean accuracy of 80.8% 4 h before onset. Figure 3b plots the receiver operating characteristic curves for 4 test-chips. The test-chips all show high area-under-the-curve (AUC) value indicating the ability to distinguish between sepsis and non-sepsis cases with high accuracy.

Figure 3c compares energy consumption of proposed RC+ANN with estimated energy of conventional technique of digitizing ECG segments and transmitting the digitized data, and digital baseline which performs feature extraction on digitized ECG segment followed by digital ANN before transmission of prediction scores. Transmission energy is assumed to be state-of-the-art 38 pJ/bit^21^, and the ADC for digitizing ECG segment is assumed to consume 5 fJ/conversion-step at 1 kHz and 12-bit resolution^22^. RC+ANN reduces energy/inference by $13\times$ compared to digital baseline at 2% loss in accuracy, and by $159\times$ compared to conventional technique. The proposed analog-to-information conversion technique reduces RF transmission by close to $5400\times$. This significant reduction in RF transmission is due to the fact that for every ECG segment the on-chip RC+ANN transmits only prediction score instead of transmitting all the digitized ECG samples in that segment. In this work, we consider non-overlapping ECG segments with 6000 samples for analysis. If the entire ECG segment is digitized and transmitted, the transmission energy needed is $2.73\,\upmu$J at 38 pJ/bit for 6000 samples digitized with 12-bit resolution. Instead of transmitting each ECG samples in the segment, transmission of RC+ANN prediction requires digitization of only the RC+ANN output. Since the RC+ANN output is differential, the prediction score is digitized to 13-bit precision, and its transmission consumes only $0.5\,\upmu$J. Thus, the $5400\times$ reduction in RF transmission is due to the reason that for each ECG segment, the proposed analog-to-information conversion techniques requires transmission of 13 bits instead of 72000 bits with naive transmission of all ECG samples.

## Discussion

### Comparison with state-of-the-art

Table 1 compares the proposed reservoir-computing model with state-of-the-art software AI models for MIMIC–III dataset. The proposed technique compares favorably with state-of-the-art using single modality sensor data source and no laboratory test results, which demonstrates feasibility of the proposed technique for at-home monitoring and is a key differentiation from state-of-the-art which requires multiple modality sensor data and/or laboratory test results. Table 2 compares efficiency in terms of tera-operations/watt (TOPS/W) of the RC+ANN with state-of-the-art in-memory computing AI accelerator macros. The proposed RC+ANN achieves competitive power efficiency as state-of-the-art AI accelerators and matrix multiplier macros even after including energy for data movement and output activations. Compared to the state-of-the-art hardware accelerators, the proposed RC+ANN circuit achieves competitive area efficiency (in terms of TOPS/W/mm$^2$), but in general SRAM based macros have an order-of-magnitude better area efficiency. This is expected since SRAM cells are fully digital and have a much higher density than the analog switched-capacitor circuits used in our design.

**Table 1:** Comparison with state-of-the-art software AI models for sepsis prediction.

	^23^	^24^	^25^	^26^	^27^	This work
	Nature Sc. Reports’19	ICHI’21	Plos One’21	Crit. Care Med. ’18	Plos One’22	
AI model	RNN$^{1}$	CNN$^{2}$	DL-ATT$^{3}$	Cox	LSTM	RC+ANN
Time-to-onset	7 h	6 h	4 h	4 h	1 h	4 h
Accuracy	−	84.7%	−	64%	−	80.8%$^{4}$
Sensitivity	0.88	0.87	0.49	0.89	0.85	0.84$^{4}$
Specificity	0.84	0.86	−	0.90	0.64	0.75$^{4}$
Vitals	10	0	7	10	6	1
Lab tests	6	13	17	30	27	0
EMR$^{5}$	0	0	3	19	3	0

$^1$Long-short term memory.

$^2$Recurrent neural network.

$^3$Attention-based deep-learning model.

$^4$Average of 4 test-chips.

$^5$Demographics and co-morbidities.

**Table 2:** Comparison with AI hardware accelerator.

Compute type	^10^	^11^	^12^	^13^	^14^	^15^	^28^	^16^	^17^	This
	VLSI’18	JSSC’20	ISSCC’19	JSSC’18	JSSC’20	ISSCC’19	TCAS1’19	ISSCC’21	ISSCC’22	work
	SRAM					Analog				
	10T1C	12T	8T	6T	8T1C	ReRAM	ELM	3T DRAM	TD+SC	RC+ANN
Process	65 nm	65 nm	55 nm	65 nm	65 nm	55 nm	65 nm	65 nm	28 nm	65 nm
Weight precision	1	1	2	8	1	1	1	4	5	1(RC)
										4–4–6
										(ANN)$$^1$$
Input precision	1	1	1	8	1	3	8	4	5	12(RC)
										10–8–8
										(ANN)$$^1$$
Efficiency TOPS/W	658$^2$	403$^2$	18.4$^2$	6.25$^2$	671.5$^2$	53.2$^2$	2.9$^2$	32.5$^2$	13.3$^2$	43.6$^3$
Norm. eff. (TOPS/W)$^4$	658	403	36.8	400	671.5	159.5	23.2	520	332.7	528$^3$
Throughput (GOPS)	9438	−	−	4.1	1638	−	−	−	100.8	0.8$^3$
Area eff. (TOPS/W/mm$^2$)	104.4	6307	5808	1143	8290	787.6	13.8	157.6	264	277.9$^3$

$^1$Precision for 2 hidden layers and output layer; $^2$One MAC is considered as 2 OPS (multiplication and addition) and does not include energy for data movement and output activations; $^3$Nonlinearity, ADC and DAC of RC are considered as 1 operation each; $^4$Normalized efficiency is given by efficiency (TOPS/W) $\times$ input precision $\times$ weight precision.

### Analog in-memory computation using switched-capacitor circuits

Energy efficiency of traditional AI computing systems are limited by communication costs of bringing together many input activations, and neuron weights, and distributing output activations in von-neumann architectures with separate memory and computing units. Wearable sensors have low energy budget which cannot accommodate conventional AI computing systems. In-memory computing (IMC) can break the von-neumann bottleneck by massively parallelizing computations and drastically reducing communication costs by performing computations using memory units. In this work, we perform analog IMC by using switched-capacitor circuits that store the ANN weights as capacitor values and intermediate results as charge on the circuit nodes. The complete vector matrix multiplications across all the neurons in each layer are performed simultaneously, and the results are stored locally in charge-domain on the shared top-plate of the capacitors. Apart from charge-domain IMC, SRAM based IMC is another popular technique for vector matrix multiplications using CMOS circuits. Compared to SRAM based IMC, switched-capacitor IMC has better linearity for vector matrix multiplications. This is because arithmetic computation through passive charge sharing/redistribution in switched-capacitor IMC is more linear and less sensitive to random variations introduced during chip fabrication than SRAM array since switched-capacitor circuits use ratios of capacitors for computation. In contrast, linearity of vector matrix multiplication using SRAM cells is fundamentally limited by nonlinear relationship between current and voltage on the bitlines where the accumulation happens, and the vector matrix multiplication results are not linear over the full dynamic range^29^. In addition, matching capacitors in switched-capacitor circuits is easier than matching transistors in SRAM cells across large IMC array. However, the trade-off of using switched-capacitor IMC is the lack of re-configurability of ANN weights encoded as capacitor values in switched-capacitor IMC circuits, whereas the ANN weights can be easily re-configured in SRAM array by re-writing new weights into the array.

### Limitations and future work

In this work, cleaned ECG signal from MIMIC–III dataset has been used to demonstrate the proposed technique of analog-to-information conversion through in-sensor AI. However, in practical at-home monitoring applications, the acquired ECG signal is likely to contain artifacts and will require analog front-end (AFE) with band-pass filtering before the ECG signal is sent to the RC+ANN combination. The AFE will consume additional power that will reduce the energy advantage of the proposed technique over the conventional method of transmitting all the sensor data. As an example, state-of-the-art AFEs for ECG sensor typically consume $1-8\times$ power of ADC^1,3,7,30^, and a similar AFE in front of our RC+ANN will result in $3.8-21\times$ reduction in energy compared to naive transmission. Hence, the in-sensor AI technique shifts the design burden for high energy efficiency from transmitter to the AFE. The AFE energy efficiency can be potentially improved through inverter based amplifier design and inverter stacking^31,32^ to reduce overall energy consumption of the sensor.

A circuit level limitation of our work is the lower area efficiency our circuit than state-of-the-art SRAM based AI circuits. This area limitation is fundamentally due to the use of switched-capacitor circuit as building blocks for matrix multiplication which has lower area efficiency than SRAM cell. The area density (in terms of fF/$\upmu$mm$^2$) of metal-on-metal capacitors used in this design does not scale as much as transistor area density, thus the area efficiency advantage of SRAM circuits over RC+ANN is likely to increase with CMOS technology scaling. Since computations in the input and reservoir layer can be nonlinear, a potential solution to improve area efficiency of the RC+ANN is to adopt SRAM arrays for matrix multiplications in the input and reservoir layers, and use switched-capacitor circuits for the ANN based read-out only where higher linearity is needed.

## Methods

### Reservoir-computer design

RC is a well-known computing paradigm that uses static nonlinearity to project the input signal to high-dimensional space, thus allowing easier separation of different input classes. No training is performed in the input or reservoir layers, and the weights are drawn from random distribution. While reservoir computing was invented almost two decades earlier and has been extensively used in the machine-learning literature, hardware implementation of reservoir computing have been mostly on optics/photonics platform with few analog silicon implementations^33–35^. In contrast to prior silicon RC, the proposed RC is based on the architecture in^36^ and does not require large capacitors to realize biological time-constants which is energy-inefficient, and does not require background calibration for analog delay elements or nonlinearity element. Output of the RC with *N* reservoir neurons can be mathematically expressed as

1 $$\begin{aligned} {\vec{R_k}[n]} = H\left( G_i \vec{W} \times \vec{X}[n] + G_f \vec{W_r} \times \vec{R_k}[n-1] \right) \end{aligned}$$

where $\vec{X}$ is analog ECG input with *D* samples, $\vec{W}$ is $N \times D$ input weight matrix, $\vec{W}$ ($D>> N$), $\vec{W_r}$ is $N \times N$ inter-connection weight matrix for the reservoir layer, $H(\cdot )$ is nonlinear activation for RC, $G_i$ is input scaling factor and $G_f$ is feedback gain. As in^36^, identity matrix is used for $\vec{W_r}$ which simplifies the hardware implementation since $\vec{W_r}$ can be realized using a single-cycle delayed feedback. The restriction on $\vec{W_r}$ is consistent with^37^ which has shown through systematic investigation that a simple reservoir architecture with sparsely inter-connected reservoir provides comparable accuracy as more complicated reservoir architectures. $G_i$ and $G_f$ and *N* are set to 0.6, 0.1 and 63 respectively to optimize prediction accuracy and ensure stability of the reservoir, $D=6000$ corresponding to 20 s ECG segments.

**Figure 4:**
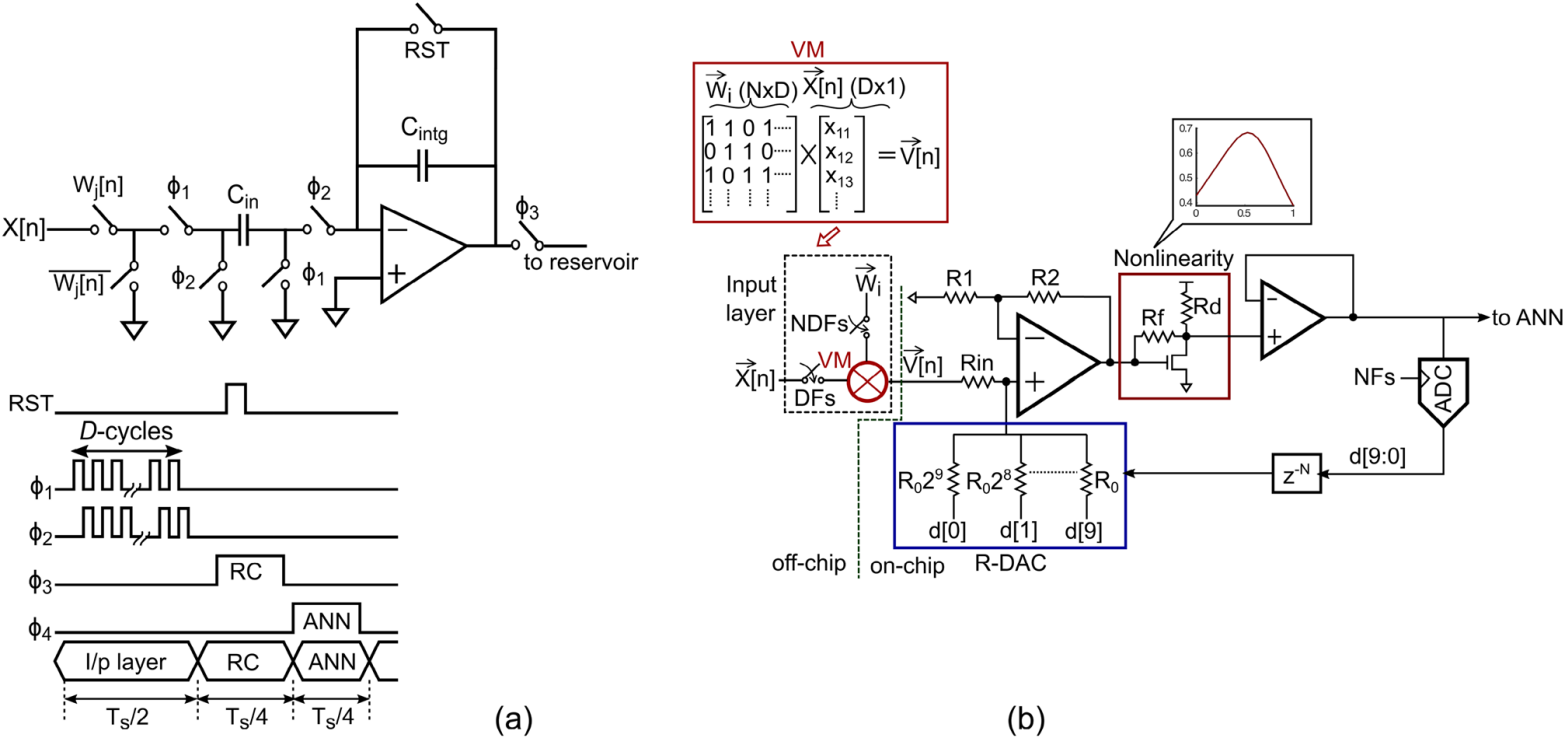
Circuit schematics for the reservoir-computer for analyzing ECG signals. (**a**) Schematic of the input layer of the reservoir showing the switched-capacitor multiplier (**b**) schematic of a single reservoir neuron.

Figure 4 shows the circuit schematics of the reservoir layer and input layer and the timing diagram. Elements of $\vec{W}$ are set to ‘0/1’ which converts matrix multiplication in the input layer to addition. Switched-capacitor (SC) integrator is used to perform charge-domain accumulation and store partial results in the feedback capacitor, $C_{intg}$ (Fig. 4a). The accumulated results from the input layer are sent to the reservoir layer shown in Fig. 4b. An operational-transconductance amplifier (OTA) is used to sum input to the reservoir layer with delayed feedback from the reservoir neuron. Output of the OTA represents the term within parenthesis in (1) and is passed through the nonlinearity $H(\cdot )$ which is implemented using a feed-forward common-source amplifier as shown in Fig. 4b. The non-linear activation function $H(\cdot )$ is based on Mackay-Glass nonlinearity. Output of the nonlinearity circuit is buffered and drives a 10-bit successive approximation register (SAR) ADC, and its delayed output is fedback to the input OTA through a resistive digital-to-analog converter (R-DAC). The reservoir layer is time-multiplexed to save on-chip area such that one physical neuron is used to realize *N* virtual neurons by operating the reservoir layer at $NF_s$ where $F_s=1/T_s$ is the frequency of operation of the RC+ANN and ECG input is sampled at $DF_s$. The ADC is used in the reservoir loop for accurate generation of *N*-cycle delay in the time-multiplexed feedback path since generation of precise analog delay is difficult in practice. The RC input layer is off-chip for this design to allow testing with different $\vec{W}$.

**Figure 5:**
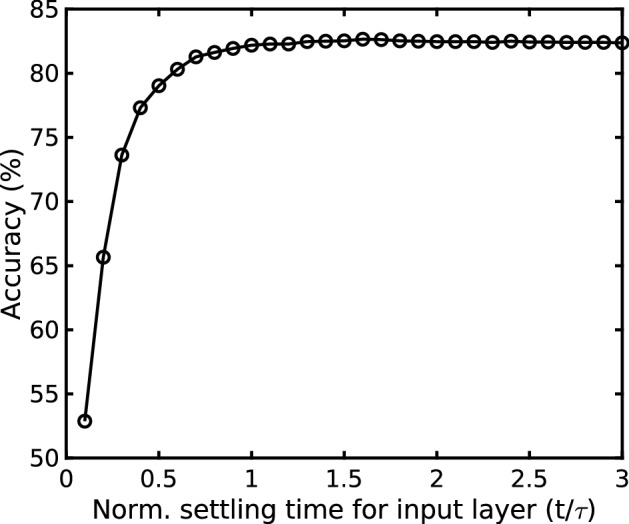
Simulated accuracy versus input layer bandwidth.

**Figure 6:**
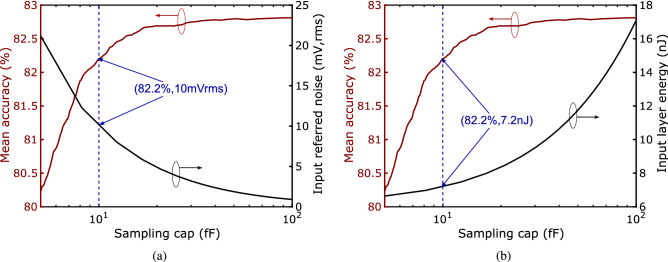
Simulated (**a**) accuracy and noise; and (**b**) energy of input layer versus sampling capacitor.

In contrast to conventional analog design, the circuits components in the RC can be nonlinear since all nonlinearity is absorbed into the reservoir dynamics. Relaxed linearity requirements allow amplifiers in the input layer and RC as well as the ADC to be low bandwidth, which results in increased nonlinearity due to slewing and incomplete settling, but reduces both noise and power. Figure 5 shows the simulated accuracy from ECG analysis as a function of settling time in the switched-capacitor input layer, and time-constant of the amplifier ($$\tau $$) is set to $$ T_s/8/D$$. Size of the sampling capacitor in the input layer is set by noise and accuracy requirements. Input-referred noise in the input layer can be shown to be

2 $$\begin{aligned} {\overline{V_{n,in}^2}} = \frac{kT}{C_{in}} \frac{1}{1-\beta } + \frac{1}{\beta }\frac{kT}{C_{eq}}\left( \frac{4}{3}\right) \left( \frac{C_{intg}}{C_{in}} \right) ^2 \end{aligned}$$

where $\beta$ is the feedback factor and $C_{eq} = C_L + (1-\beta )C_{intg}$, $C_L$ is the load capacitor. Keeping the feedback capacitor $C_{intg}$ fixed to 400fF and load capacitor of 100fF, the sampling capacitor $C_{in}$ is swept and the RC+ANN accuracy and energy of the input layer are plotted in Fig. 6. Based on the simulation results, $C_{in}$ is set to 10fF.

**Figure 7:**
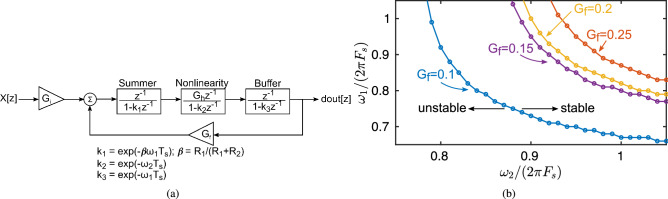
(**a**) Linearized model of the RC (**b**) stability contours.

The lower bound on bandwidth of amplifiers in the reservoir layer is set by stability requirements. Since the reservoir is strongly nonlinear, the RC loop has to be linearized around its operating point to theoretically analyze stability. The worst-case scenario from stability perspective occurs when the RC loop has the highest gain, corresponding to the highest gain of the nonlinearity function $H(\cdot )$ that occurs for the smallest input seen by the nonlinearity circuit. The highest possible gain for $H(\cdot )$ is found through simulations for different values of feedback gain, $G_f$. Figure 7a shows the discrete-time, linearized model of the RC with $G_h$ denoting gain of $H(\cdot )$. The summing amplifier and the unity-gain buffer in Fig. 4b use the same OTA with unity-gain bandwidth of $\omega _1$ and feedback factor of the summing amplifier is $\beta$, and 3-dB bandwidth of the nonlinearity circuit is $\omega _2$. Stability of the RC is analyzed by finding the roots of (3)

3 $$\begin{aligned} {1+\frac{z^{-3}}{\left( 1-k_1z^{-1}\right) \left( 1-k_1z^{-1}\right) \left( 1-k_1z^{-1}\right) } = 0 } \end{aligned}$$

Figure 7b plots stability contours versus normalized values of $\omega _1$ and $\omega _2$ as a function of $\beta$. The stable region shrinks as $G_f$ increases, and $\omega _1$, $\omega _2$ reduce. $\omega _1$ and $\omega _2$ are set to $2\pi \times 0.9F_s$ ($2\pi \times 0.9NF_s$ after time-multiplexing) for $G_f=0.1$ to ensure a wide stability margin.

### ANN model training and circuit design

**Figure 8:**
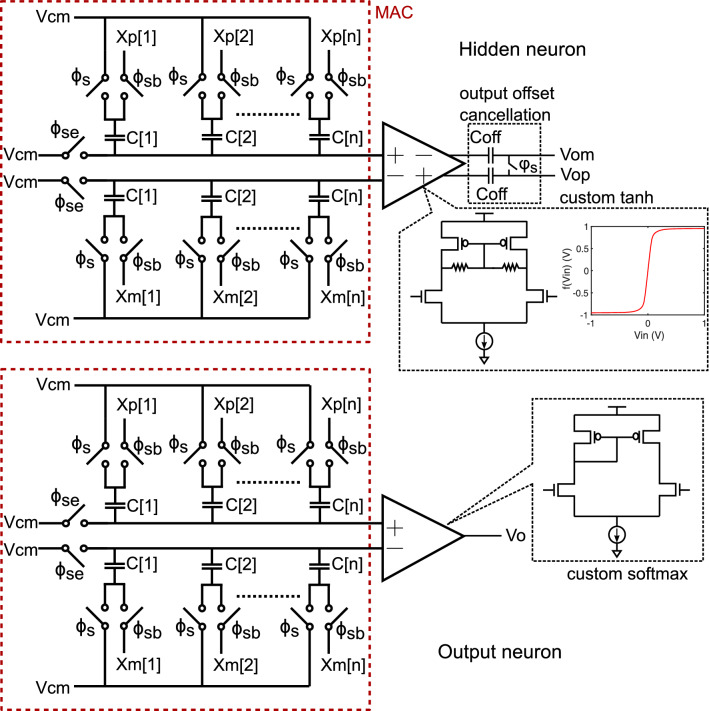
Circuit schematic of custom hidden and output neurons.

The ANN has 20 neurons in the first hidden layer, and 6 neurons in the second hidden layer. The hidden layers use custom tanh activation function, while the output layer uses a custom softmax activation function. The voltage output of the softmax function is compared with a threshold voltage to generate the ANN decision, i.e., non-sepsis/sepsis. The activation circuits are designed using single-stage, common-source differential amplifiers as shown in Fig. 8. The fully differential amplifiers in the hidden layers use output offset cancellation technique to reduce amplifier offset. Offset in the output layer is removed through foreground calibration as described later. The custom analog activation functions resemble their ideal, mathematical counterparts, but are not exactly the same. To ensure good matching between software ANN model and IC measurements, we use a hardware-software co-design methodology in which amplifier transfer curves, and their derivatives, are used to train the ANN model iteratively^38^. Stochastic gradient descent is used to optimize the ANN model by minimizing the loss function at each epoch. Once the ANN is fully trained, the model weights are encoded as capacitor values in the SC-CIM. The ANN weights are quantized to 4-bit in the hidden layers, and 6-bit in the output layer. The weight quantization is done during the training iterations to minimize effect of quantization error. A 4fF unit capacitor is used to realize an LSB weight in the SC-CIM without degrading ANN accuracy due to capacitor mismatch.

## Data Availability

The dataset used in the current study is publicly available in the MIMIC-III database (https://physionet.org/content/mimiciii/1.4/). MIMIC-III integrates de-identified, comprehensive clinical data of patients admitted to the Beth Israel Deaconess Medical Center in Boston, Massachusetts, and makes it widely accessible to researchers internationally under a data use agreement^39^.
